# Clinical, Laboratory, and Therapeutic Characteristics of Visceral Leishmaniasis with Emphasis on Immune Status: A Multicentre Cohort Study in Greece

**DOI:** 10.3390/pathogens15020141

**Published:** 2026-01-28

**Authors:** Aristos Aristodimou, Achilleas Gikas, Maria Antoniou, Karolina Akinosoglou, Nikolaos Partalis, Angelos Pefanis, Periklis Panagopoulos, Charalambos Christofidis, Evangelos I. Kritsotakis

**Affiliations:** 1 Division of Internal Medicine, School of Medicine, University of Crete, 70013 Heraklion, Greece; gikas.achilles@gmail.com; 2 Parasitology and Medical Entomology Unit, School of Medicine, University of Crete, 70013 Heraklion, Greece; antoniou@uoc.gr; 3 Department of Internal Medicine, University General Hospital of Patras, 26504 Rio, Greece; akin@upatras.gr; 4 First Department of Internal Medicine, General Hospital of Chania, 73100 Chania, Greece; 5 Department of Medicine and 1st Department of Infectious Diseases, General Hospital for Thoracic Diseases “Sotiria”, 11527 Athens, Greece; 6 Second Department of Internal Medicine, University General Hospital Alexandroupolis, Democritus University of Thrace, 68100 Alexandroupolis, Greece; ppanago@med.duth.gr; 7 Second Department of Internal Medicine, General Hospital of Volos, 38222 Volos, Greece; 8 Laboratory of Biostatistics, School of Medicine, University of Crete, 70013 Heraklion, Greece; e.kritsotakis@uoc.gr

**Keywords:** visceral leishmaniasis, immunosuppression, Mediterranean region, Greece, *Leishmania infantum*, treatment outcome

## Abstract

Visceral leishmaniasis (VL) is an endemic zoonotic disease in southern Europe with increasing clinical relevance among immunocompromised populations; however, detailed clinical data remain scarce. This retrospective multicentre cohort study analysed patients with confirmed VL treated at seven hospitals in Greece over a 26-year period. Clinical, treatment, and outcome data were collected with a minimum follow-up of 18 months to assess cure, treatment failure, relapse, and mortality. A total of 144 patients were enrolled (59% male; mean age 41.8 years, range 0.1–84 years), most of whom were Greek nationals (85%) and resided in rural areas (61%). Fever was the primary reason for hospital admission in 95% of patients. At diagnosis, 42 patients (29%) were immunocompromised. These patients were significantly older than immunocompetent individuals and more likely to present with diarrhoea and arthralgia, whereas hepatomegaly was less frequent. Liposomal amphotericin B was administered to 90% of patients. Treatment failure occurred in 14 patients (10%) and was significantly associated with immunosuppression and leukaemia. Relapse within 18 months occurred in 5.5% of patients. Overall mortality was relatively low (7 patients, 5%), with one death directly attributable to VL. This study demonstrates that VL remains endemic in Greece, affects patients across all age groups, and is primarily autochthonous. Immunosuppression is associated with distinct clinical features and poorer treatment outcomes in VL, underscoring the need for heightened clinical vigilance, combined diagnostic approaches, and extended follow-up in vulnerable populations.

## 1. Introduction

Visceral leishmaniasis (VL) is a potentially life-threatening parasitic disease caused by *Leishmania* spp. and remains one of the most important neglected tropical diseases worldwide [1]. Despite its geographically focal distribution, VL continues to pose a significant public health concern in the Mediterranean basin, including Greece [2].

In southern Europe, VL is caused almost exclusively by *Leishmania infantum*, transmitted through the bite of infected *Phlebotomus* sandflies, with domestic dogs serving as the primary reservoir hosts [3,4]. Although considered hypoendemic compared with high-burden regions, VL persists in Greece, where autochthonous human cases and widespread canine seropositivity indicate ongoing zoonotic transmission [5].

In recent decades, the number of immunocompromised individuals has increased owing to population ageing, HIV infection, malignancies, organ transplantation, and the expanding use of immunosuppressive therapies, including corticosteroids, azathioprine, methotrexate, chemotherapeutic agents, and biological agents. These patients may display atypical clinical or laboratory features and often experience poorer treatment responses, higher relapse rates, and increased mortality [6].

National surveillance data classify Greece among countries reporting 1–50 new VL cases annually [2]. Nevertheless, country-specific clinical and epidemiological data remain limited. Georgiadou et al. reported the clinical, laboratory, and therapeutic characteristics of VL in a 7-year, two-centre cohort, underscoring the diagnostic and therapeutic challenges in the Greek setting [7]. However, long-term real-world data from Mediterranean endemic regions remain scarce, particularly regarding differences between immunocompetent and immunosuppressed patients.

Several cohorts from Mediterranean countries have provided important insights into the epidemiology, clinical presentation, and outcomes of VL, particularly among immunocompromised populations [6,7]. However, reported outcomes vary considerably across studies, possibly reflecting heterogeneity in study design, follow-up duration, and patient characteristics. Consequently, additional multicentre real-world studies from Mediterranean endemic settings are needed to better contextualise disease presentation and outcomes.

Immunosuppression is increasingly recognised as a key modifier of VL, influencing disease susceptibility, clinical presentation, diagnostic performance, treatment response, and risk of relapse. However, real-world data directly comparing immunocompetent and immunocompromised patients within the same endemic setting remain limited. We therefore hypothesised that immune status significantly affects disease presentation and prognosis.

On this basis, we conducted a 26-year retrospective multicentre study of VL cases diagnosed and treated in seven hospitals in Greece, primarily in Crete. We aimed to describe the clinical, laboratory, and treatment outcome characteristics of VL and to compare these findings between immunocompetent and immunosuppressed patients, to inform clinical practice and improve prognostication. Compared with previously published Mediterranean cohorts, this study provides real-world data from seven hospitals in Greece and incorporates an extended follow-up of 18 months after treatment completion, allowing assessment of relapses, particularly among immunocompromised patients.

## 2. Materials and Methods

All patients with confirmed VL who were diagnosed at one of seven public hospitals in Greece between January 1994 and December 2019 were included in the study. The study centres were the University Hospital of Heraklion, the “Venizeleio-Pananeio” General Hospital of Heraklion, the General Hospital of Chania, the University General Hospital of Patras, the “Sotiria” General Hospital of Athens, the General Hospital of Volos, and the General Hospital of Alexandroupolis. The Institutional Review Boards of all participating hospitals approved the study. The extended study period was selected to maximise case accrual in a low-incidence endemic setting and to allow evaluation of long-term real-world patterns of disease presentation and outcomes across different eras of clinical practice.

Electronic and written patient records were reviewed for demographic characteristics, comorbidities, laboratory parameters, diagnostic methods, treatment, and outcomes. Clinical manifestations and laboratory parameters were recorded at the time of hospital admission for VL, prior to initiation of antileishmanial therapy. All adult and paediatric patients were eligible for inclusion, and no cases were excluded solely on the basis of age, atypical clinical presentation, or incomplete medical records, provided that the predefined diagnostic criteria for confirmed VL were fulfilled.

An index patient was defined, according to internationally accepted criteria, as a confirmed VL case presenting with compatible clinical and laboratory manifestations (two or more of the following: persistent fever > 38 °C, hepatosplenomegaly, substantial weight loss, anaemia, leukopenia, polyclonal hypergammaglobulinemia, or lymph node enlargement), together with confirmatory evidence from at least two diagnostic modalities, including serology, demonstration of the parasite in a tissue smear, or molecular detection by PCR, depending on availability at the time of diagnosis [8,9].

Diagnostic practices were broadly consistent across participating centres but evolved over the long study period. In earlier years, diagnosis relied primarily on serology and parasitological confirmation, whereas molecular methods (PCR) became increasingly available later. Historical cases were classified according to the diagnostic standards applicable at the time of diagnosis. Temporal and inter-centre variability, therefore, reflected differences in diagnostic availability over time.

Patients were classified as immunosuppressed if they had: (a) a history of active malignant disease (haematological malignancy or solid tumour); (b) an inherited or acquired immunodeficiency condition (e.g., splenectomy, HIV infection); or (c) were receiving immunosuppressive therapy (e.g., corticosteroids, azathioprine, methotrexate, chemotherapeutic agents, or biological agents). Regarding corticosteroids, immunosuppression was defined as a daily dose of ≥5 mg of prednisone or equivalent for at least 30 consecutive days [7]. These conditions were analysed as a single group to allow an exploratory comparison of clinical characteristics, laboratory findings, and outcomes in patients with impaired immune status, rather than to imply a uniform biological effect across conditions. We acknowledge the clinical heterogeneity of this population; however, subgroup analyses were not performed due to the limited number of immunocompromised patients and outcome events, which would have resulted in insufficient statistical power and unreliable estimates.

Haematological abnormalities were defined using standard clinical thresholds. Haemoglobin < 11.5 g/dL in females and <13 g/dL in males defined anaemia; a leukocyte count < 4 × 10^9^/L defined leukopenia; and a platelet count < 150 × 10^9^/L defined thrombocytopenia [10]. Laboratory values were recorded as reported by each participating centre and interpreted according to local reference ranges at the time of testing; for analytical purposes, uniform clinical thresholds were applied to ensure comparability across centres.

Clinical response was evaluated at completion of therapy and classified as either cure or failure. Cure was defined as defervescence accompanied by restoration or clear improvement of laboratory parameters, and/or significant reduction in splenomegaly. Failure was defined as persistence or worsening of clinical or laboratory abnormalities, together with persistence of the *Leishmania* parasite in tissues after completion of treatment, including relapse [8,11].

The patients were followed up for 18 months after completion of antileishmanial therapy, and relapse was defined as the recurrence of VL-compatible signs or symptoms within 18 months of initial clinical cure, consistent with published data indicating that a significant proportion of relapses may occur beyond 6–12 months of follow-up [12,13,14]. Initial cure was defined as clinical cure, based on resolution of fever and improvement or normalisation of clinical and laboratory parameters; parasitological confirmation of cure was not systematically required and was performed only when clinically indicated. The 18-month follow-up was completed retrospectively for all patients by reviewing the medical records and electronic patient files to identify readmission or re-evaluation at the hospital where the initial treatment was administered.

Categorical data were presented as frequency counts and percentages and compared across patient groups using Pearson’s chi-squared test or Fisher’s exact test when sample sizes were small (resulting in expected frequencies < 5). Continuous data were summarised as mean and standard deviation and were compared between groups using the t-test. When sample sizes were small (*n* < 25 per group) and distributions were skewed or contained extreme outliers, continuous data were summarised as median and interquartile range (IQR) and the Wilcoxon–Mann–Whitney U test was used for between-group comparisons. Comparisons were made between immunocompetent and immunocompromised patients, and between groups defined by cure and treatment failure. A two-tailed *p*-value < 0.05 was considered to indicate statistical significance. Complete case analyses were performed, and missing data due to an inability to retrieve relevant information were noted as “unknown” in the tables of results. Due to the small effective sample sizes (i.e., small numbers of immunocompromised patients and treatment failures), multivariable regression analysis was not possible. Data were processed and analysed using STATA v.19 (StataCorp, College Station, TX, USA).

## 3. Results

**Figure 1:**
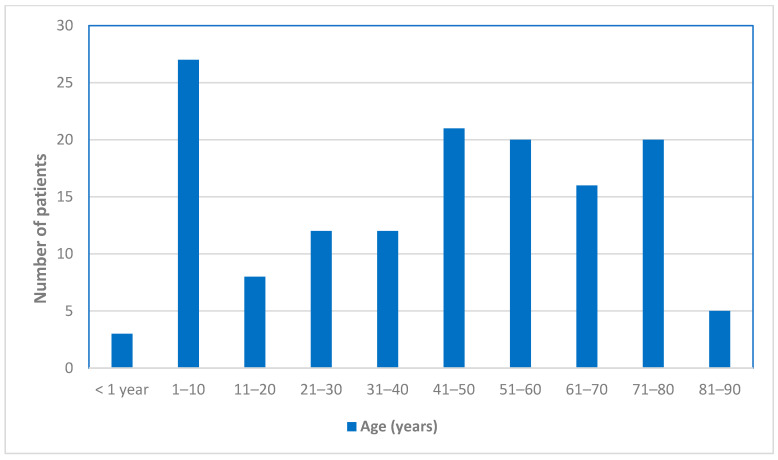
Distribution of patients with visceral leishmaniasis according to age.

During the 26-year study period, 142 patients who met the criteria for confirmed VL and two patients with concomitant VL and cutaneous leishmaniasis (CL) were identified. The mean ± SD age of the patients was 41.8 ± 26.5 years, and 59% were male. The age distribution of the patients is shown in Figure 1. Of the 144 patients included, 86 were diagnosed at the University Hospital of Heraklion, 23 at the Venizeleio–Pananeio General Hospital of Heraklion, 6 at the General Hospital of Chania, 17 at the University General Hospital of Patras, 6 at the “Sotiria” General Hospital of Athens, 3 at the General Hospital of Volos, and 3 at the General Hospital of Alexandroupolis. The distribution of cases by time period was 37 (26%) in 1994–2010, 40 (28%) in 2011–2014, and 67 (46%) in 2015–2019.

One hundred and twenty-three patients (85%) were Greek nationals, and 88 patients (61%) resided in rural areas. Regarding exposure to dogs, among patients for whom relevant information was available, 61% were dog owners, and 21.7% of these dogs had been diagnosed with leishmaniasis. Additionally, 31.8% of patients reported stray dogs near their residences.

The main clinical, physical examination, and laboratory findings are presented in Table 1. At diagnosis, 102 patients (70.8%) were immunocompetent, whereas 42 patients (29.2%) were considered immunocompromised. Of the latter, 29 were receiving immunosuppressive therapy, five had HIV infection, four had haematological malignancies, two were asplenic, one had autoimmune disease combined with chronic kidney failure, and one had polyclonal hypogammaglobulinemia.

The median duration of symptoms at admission, among patients for whom this information was available, was 25 days (interquartile range: 13.5–45 days). Overall, 10 patients (7%) reported low-grade fever, whereas 45 (31%) had a fever of 38–39 °C and 73 (51%) had a fever ≥ 39.1 °C. For nine patients (6%), information on the maximum temperature was missing, and seven patients (5%) did not report fever. Notably, fever was the main presenting complaint leading to hospital admission in 110 patients (76%). One patient was recorded as completely asymptomatic, and VL was diagnosed during investigation for anaemia and neutropenia. Overall, fever was present in the vast majority of patients, was the most common reason for hospital admission, and was frequently high-grade.

Additionally, at the time of diagnosis, 16 patients (11%) had concurrent infections, including pneumonia (*n* = 2), bacteraemia (*n* = 2), upper respiratory tract infection (*n* = 2), and urinary tract infection (*n* = 2). Single cases of *Mycobacterium avium* infection, rotavirus infection, and *Salmonella typhi* infection were observed. Q fever was diagnosed in one patient and influenza in another. Two patients had cytomegalovirus (CMV) viraemia (without end-organ disease) detected at the time of diagnosis, one of whom also had *Klebsiella pneumoniae* meningitis. Finally, HIV infection with *Pneumocystis jirovecii* pneumonia was diagnosed in one patient. Of note, seven patients (5%) developed hemophagocytic lymphohistiocytosis (HLH).

A range of diagnostic methods was used to diagnose VL. One hundred twenty-one patients (84%) had positive serology, while 68 patients (47%) had positive direct smears (66 bone marrow, one lymph node, and one spleen specimen). Polymerase chain reaction (PCR) specific for *Leishmania* species in peripheral blood and/or bone marrow was positive in 71 patients (49%). In all cases in which species identification was performed, *Leishmania infantum* was exclusively identified (29 cases).

One hundred thirty patients (90.2%) were treated with liposomal amphotericin B (L-AMB), five patients (3.5%) received meglumine antimoniate, while three patients declined treatment. One patient died from septic shock before treatment initiation. Information regarding the type of treatment administered was unavailable for five patients.

Treatment-related adverse events occurred in 22 patients (15.2%). Reported side effects associated with L-AMB included rigours, deterioration of renal function, allergic reactions, hypokalaemia, arthralgia, and transaminase elevation. Notably, one patient treated with L-AMB developed pleural effusion, and another experienced a Jarisch–Herxheimer reaction. Among patients treated with meglumine antimonate, electrocardiographic repolarisation abnormalities were recorded.

Two patients did not achieve complete fever remission following treatment with L-AMB. One of these patients responded well to etoposide therapy for HLH, while the other became afebrile after treatment for concomitant acute myeloid leukaemia.

Eight patients (5.5%) experienced relapse within 18 months. All eight received a second cycle of L-AMB, and seven were cured. One patient did not become afebrile after retreatment and was subsequently diagnosed with concomitant non-Hodgkin lymphoma; this patient died due to septic shock caused by multidrug-resistant *Klebsiella pneumoniae*.

Overall, seven patients died during the first 12-month follow-up period (crude mortality). One death was directly and clearly attributable to active VL, four were attributed to bacterial infections, and one was attributed to acute myeloid leukaemia. For one patient, despite a thorough review of the available clinical documentation, the cause of death remained undetermined.

Table 1 also contrasts the epidemiological, clinical, and laboratory characteristics of the patients according to their immune status. A statistically significant difference in age distribution was observed: the mean age of immunocompetent patients was 35.3 ± 25.8 years, compared with 57.5 ± 21.2 years in immunocompromised patients (*p* < 0.001). Significantly more males were immunocompetent than immunocompromised (65% versus 45%, *p* = 0.031). Regarding clinical manifestations, statistically significant differences were observed for arthralgia and diarrhoea, both of which were more prevalent among the immunocompromised patients. In addition, hepatomegaly was observed in 62% of immunocompetent patients, compared with only 33% of immunocompromised patients. It is noteworthy that, although the differences were not statistically significant, consistent trends were observed in parameters such as weakness, symptom duration, and fever duration, all of which were more frequent or longer among immunocompromised patients.

Most patients responded successfully to treatment, but 14 patients (10%) experienced treatment failure. Table 2 presents the univariate analysis of factors associated with treatment outcomes in VL. A statistically significant association was observed with immune status: 57% of patients who experienced treatment failure were immunocompromised, compared with only 26% of those who responded favourably (*p* = 0.027). Additionally, treatment failure, as opposed to cure, was associated with a significantly higher prevalence of leukaemia (21% vs. 0%; *p* < 0.001).

## 4. Discussion

In this 26-year multicentre study from Greece, the clinical, laboratory, and therapeutic characteristics of 144 patients with VL were reviewed, and factors associated with disease presentation and treatment outcome, with particular emphasis on immune status, were identified. By integrating long-term real-world data with detailed stratification by immunocompetence, this study provides clinically relevant insights into the contemporary epidemiology and behaviour of VL in a Mediterranean endemic setting.

The distributions of patients across the study centres and the predominance of cases diagnosed in Crete may reflect a combination of epidemiological and healthcare-related factors. Crete is a well-recognised endemic region for VL in Greece, with sustained zoonotic transmission and evidence of increasing incidence reported over time [5,15]. The regions of Heraklion and Chania constitute long-standing endemic foci for VL [15]. In addition, tertiary referral centres on the island serve as major diagnostic and treatment hubs for suspected or complicated cases, resulting in increased case ascertainment. Greater clinical awareness and diagnostic expertise in endemic regions may further contribute to the higher number of identified cases, a pattern that has been described in other Mediterranean settings [1,3].

The age distribution of this cohort was broad, ranging from infancy to advanced age, with a notable accumulation of cases both in childhood and among older adults. This pattern reflects a dual epidemiology of zoonotic VL in southern Europe, where transmission affects children as well as older individuals, the latter often burdened by comorbidities or immunosuppression [3,5,6]. This is in contrast in regions such as the Indian subcontinent (India, Bangladesh, and Nepal) and East Africa (including Sudan and Ethiopia), VL is more commonly reported among younger age groups [1,3,8,9]. Immunocompromised patients were significantly older than immunocompetent individuals, underscoring the contribution of age-related conditions, malignancy, and immunosuppressive therapies to VL susceptibility in contemporary European cohorts. Male predominance was observed overall, but it was less pronounced among immunocompromised patients, possibly reflecting the sex distribution of underlying conditions associated with immunosuppression rather than differences in exposure to *Leishmania*. Residential status and nationality did not differ between groups, a finding consistent with ongoing endemic transmission of VL in Greece rather than predominantly imported disease, as previously reported [5,7].

Fever was nearly universal and constituted the primary reason for hospital admission. Clinically meaningful differences emerged when patients were stratified by immune status: immunocompromised patients more frequently reported diarrhoea and arthralgia, whereas hepatomegaly was significantly less common. These findings are consistent with previous reports indicating that VL in immunocompromised patients may present with less prominent organomegaly and a higher prevalence of non-specific systemic manifestations [6,16,17]. In addition, several other features—including weakness, pancytopenia, vomiting, and peripheral oedema—showed borderline associations with immune status. The latter agree with previously described associations in leishmania cohorts in Europe [6,16,17], but were not statistically significant, and considering the limited sample sizes in this study, they cannot be regarded as confirmatory evidence for a distinct clinical phenotype.

Several factors may explain the observed differences between immunocompetent and immunocompromised patients. Impaired cell-mediated immunity is considered central to the pathogenesis of VL and, in immunocompromised individuals, this may substantially affect parasite control and host inflammatory responses, contributing to atypical or non-classical clinical presentations [1,6,9]. In this population, defective cellular immune responses (including impaired macrophage-mediated parasite killing) are thought to contribute to deviations from the typical disease phenotype and to less characteristic patterns of organ involvement, compared with immunocompetent hosts [1,6,14]. In addition, advanced age, comorbidity burden, and the use of immunosuppressive therapies may further modulate disease expression and delay immune recovery [6,16,17], thereby increasing the risk of treatment failure or relapse [6,13,14]. Differences in diagnostic performance—particularly reduced sensitivity of serological assays in patients with impaired immunity—may also influence disease recognition and management in this population [6,9,14].

Haematological abnormalities were frequent and severe across the cohort. Anaemia, leukopenia, thrombocytopenia, and pancytopenia were present in the majority of patients, without significant differences between immunocompetent and immunocompromised groups, emphasising that profound cytopenias are a hallmark of VL itself rather than a discriminator of immune status [1,9]. Polyclonal hypergammaglobulinemia was observed in approximately 70% of cases, consistent with chronic immune activation induced by *Leishmania* infection. Autoimmune markers were frequently positive, although data were often missing, reflecting the polyclonal immune activation associated with VL and potentially contributing to diagnostic complexity, particularly in patients initially evaluated for autoimmune or haematological disorders [1,6,9].

HLH was identified in 5% of patients. Although uncommon, this complication has important clinical implications. VL-associated HLH remains underdiagnosed in adults because its manifestations overlap substantially with those of VL itself and with haematological malignancies. In this context, persistent fever or cytopenias after antiparasitic therapy should not automatically be interpreted as treatment failure but may reflect ongoing hyperinflammation or occult malignancy [18,19].

The observed clinical and laboratory profiles are directly relevant to everyday clinical practice. Fever and cytopenias were very common and remain key features prompting diagnostic evaluation for VL in endemic settings [1,4,8,9]. The high prevalence of pancytopenia underscores the importance of considering VL in the differential diagnosis of febrile patients with haematological abnormalities, including in European and Mediterranean settings [1,4,8,9]. This is particularly relevant for immunocompromised individuals, in whom VL is increasingly recognised and may present atypically [6,12,14,16,17]. Differences in symptom profiles between immune groups, including more variable organomegaly and more non-specific systemic manifestations among immunocompromised patients, highlight the risk of delayed or missed diagnosis in this population [6,12,14,17]. Awareness of these atypical presentations may facilitate earlier recognition and timely initiation of appropriate therapy [8,9]. Furthermore, laboratory abnormalities such as severe cytopenias and hypergammaglobulinemia reflect disease severity and systemic immune activation and may assist clinicians in assessing disease burden and monitoring response to treatment [1,4,8,9].

Diagnosis relied on a multimodal approach combining serology, parasitological methods, and molecular testing, in accordance with international recommendations [8,9]. Nearly half of the patients required parasitological or PCR confirmation, underscoring the limitations of serology alone, particularly in immunocompromised hosts. Reduced sensitivity of antibody-based assays in patients with impaired cellular immunity has been reported in several studies, supporting the diagnostic strategy applied in the present study [16,20].

L-AMB was the predominant treatment and was generally well tolerated. The duration of treatment depended primarily on whether the patient was immunocompetent or immunocompromised and was predefined according to international guidelines [8]. Relapse within 18 months was observed in 5.5% of patients. The prolonged follow-up period strengthens the validity of this estimate, as several European studies have demonstrated that a significant proportion of relapses—especially among immunocompromised patients—occur beyond the first year after apparent cure [12,14,21].

The extended study period allows consideration of temporal patterns in the diagnosis and management of VL. Over the 26-year observation period, diagnostic availability and therapeutic strategies evolved, reflecting broader changes in clinical practice and healthcare infrastructure. In earlier years, diagnosis relied predominantly on serology and parasitological confirmation, whereas molecular methods became increasingly available later. Similarly, antileishmanial treatment strategies changed over time, with L-AMB becoming the predominant therapy in more recent years, in line with international recommendations. These changes may have influenced disease recognition, treatment response, and follow-up practices across different eras. Nevertheless, the core clinical challenges of VL—particularly among immunocompromised patients—remained consistent throughout the study period, supporting the relevance of the observed patterns to contemporary clinical practice.

Analysis of factors associated with treatment outcome identified immune status as a key determinant. Immunocompromised patients accounted for more than half of treatment failures, despite representing less than one-third of the cohort. The borderline association observed for HIV infection further supports existing evidence that HIV-related immunosuppression is associated with poorer outcomes and higher relapse rates, even in the era of L-AMB [16,21,22,23,24]. A single death was directly attributable to VL in this study. Most deaths resulted from bacterial infections or underlying disease, highlighting that prognosis in contemporary European VL cohorts is primarily driven by host factors and comorbidities rather than uncontrolled parasitic infection alone [6,7,24]. Given the absence of multivariable adjustment, these findings should be interpreted as associations and may be influenced by age, comorbidity burden, or treatment era rather than reflecting a direct causal effect of immune status.

Comparable long-term cohorts from other Mediterranean countries have reported heterogeneous outcomes, likely reflecting differences in study design, follow-up duration, population structure, and the proportion of immunocompromised patients. Studies from Spain, Italy, and France indicate that relapse and mortality are more frequent among immunocompromised individuals—particularly those with HIV infection, haematological malignancies, or solid organ transplantation—while outcomes in immunocompetent patients treated with L-AMB are generally favourable [6,12,14,21,22,23,24]. Furthermore, several European cohorts have highlighted that mortality is often driven by host comorbidities and secondary infections rather than uncontrolled parasitic disease itself [6,7,21,22,23,24]. Within this context, the relapse and mortality rates observed in the present Greek cohort are broadly comparable to those reported in other Mediterranean settings and reinforce the persistent vulnerability of immunocompromised patients despite contemporary therapeutic strategies.

Although the present findings derive from a Mediterranean endemic setting, several observations align with reports from high-burden regions in Latin America, Asia, and East Africa. In these settings, VL is similarly characterised by substantial clinical heterogeneity, frequent diagnostic challenges, and poorer outcomes among immunocompromised or comorbid patients [1,3,6,8,9]. However, important differences must be acknowledged. In contrast to regions with intense transmission, where VL predominantly affects young children and is often associated with malnutrition and delayed access to care, European cohorts—including the present study—are characterised by an older patient population, a higher prevalence of immunosuppression, and greater access to advanced diagnostic modalities and L-AMB [1,2,3,6]. These epidemiological and healthcare system differences are likely to influence disease presentation, relapse risk, and mortality, underscoring that findings from Mediterranean cohorts should be interpreted within their specific clinical and geographic context rather than extrapolated directly to high-incidence regions.

In Greece and other southern European countries, VL occurs in rural, urban and peri-urban settings, reflecting the widespread distribution of *L. infantum* and its primary reservoir, the domestic dog [3,5]. Previous studies have demonstrated that transmission is not confined to rural environments and that urbanisation has not eliminated zoonotic risk [3,5]. These findings are consistent with the present study’s results, further supporting the notion that VL remains a relevant public health concern across both rural and urban settings. Canine infection plays a central role in maintaining transmission, and high levels of canine seropositivity have been reported in endemic areas, including Greece [5]. This observation highlights the importance of targeting both human disease and canine reservoirs in Mediterranean endemic settings [1,3,5].

These findings have important implications for future surveillance, diagnostic strategies, and treatment policies in Mediterranean endemic settings. The broad age distribution and the substantial proportion of immunocompromised patients indicate that surveillance systems should focus on both paediatric and adult populations, including recognised high-risk groups (i.e., patients with malignancy, HIV infection, or receiving immunosuppressive therapies) [1,3,6]. From a diagnostic perspective, the frequency of atypical clinical presentations and the reduced reliability of serological tests in immunocompromised hosts support the continued use of combined diagnostic approaches, integrating serology with parasitological and molecular methods where available, to improve diagnostic accuracy and reduce delays [8,9,20]. Regarding treatment, the higher rates of treatment failure and relapse observed among such patients underscore the need for tailored therapeutic strategies and closer and prolonged post-treatment follow-up in this population, in line with existing evidence from European cohorts [6,12,16,21]. Collectively, these observations support strengthening integrated surveillance frameworks, enhancing diagnostic capacity, and implementing adaptive treatment policies to address the evolving epidemiology of VL in southern Europe [1,3,5].

This study has limitations inherent to its retrospective design, including missing data for certain variables and the absence of a multivariable analysis, which limits assessment of independent predictors of outcome. Multivariable analyses were not performed due to the limited number of immunocompromised patients and outcome events, which would likely have resulted in unstable models with a high risk of overfitting, particularly given potential confounding by age, comorbidity burden, and calendar time period. Variability in diagnostic data reflects the long retrospective study period during which diagnostic availability and therapeutic practices evolved and may have influenced case detection and management. The long study period introduces temporal heterogeneity in diagnostic approaches, therapeutic regimens, and overall clinical management. Nevertheless, this variability also reflects real-world practice in an endemic setting, and the core clinical challenges of VL—particularly in immunocompromised patients—remain relevant to contemporary care. In addition, data on autoimmune markers were not available for all patients, which limits the interpretability of observations related to autoimmune manifestations and precludes firm conclusions in this regard.

Due to its retrospective design, follow-up relied on the review of medical records and electronic patient files from the hospital where initial treatment was administered. Consequently, it is theoretically possible that a small number of patients experienced relapse but sought care at another healthcare facility, potentially leading to a minor underestimation of relapse rates. Additional scenarios include death from unrelated causes outside the hospital setting before completion of the 18-month follow-up period, or, particularly among migrant populations, return to the country of origin without subsequent re-evaluation. Nevertheless, we are confident that the likelihood of missing a substantial number of patients is very low. In Crete, where most cases were diagnosed, the regional reference diagnostic laboratory for VL is based at the Department of Medicine, University of Crete. As a result, a suspected relapse typically prompts repeat diagnostic testing through the same referral pathway, making unrecognised relapse unlikely. Moreover, patients with recurrent symptoms would be expected to return to the same hospital where they initially received treatment for disease recurrence or reassessment, given the need for specialised care. Finally, severe or recurrent VL typically requires specialised inpatient management, further limiting the likelihood of unrecorded relapse or disease-related mortality outside the established referral network.

Analyses were not stratified by calendar period, as further subdivision of the cohort would have markedly reduced sample size and statistical power. Accordingly, associations between immune status and outcomes may be influenced by era-specific diagnostic and therapeutic practices. The retrospective inclusion of hospitalised cases may introduce some degree of selection bias, potentially under-representing milder forms of disease managed in outpatient settings. Furthermore, although uniform analytical definitions were applied, inter-centre variability in diagnostic work-up and clinical management cannot be fully excluded. Finally, as this cohort reflects real-world practice in Greece, caution is warranted in extrapolating these findings to non-Mediterranean settings or regions with different epidemiological patterns.

Nevertheless, the long observation period, multicentre design, structured relapse definition, and detailed stratification by immune status provide robust and clinically relevant real-world insights into VL in a Mediterranean endemic setting.

In conclusion, VL remains a clinically significant infection in Greece, affecting patients across a broad age spectrum. Immunosuppression is associated with distinct clinical features and markedly worse treatment outcomes, particularly in patients with HIV infection and haematological malignancies. Awareness of atypical presentations, systematic use of combined diagnostic modalities, and prolonged post-treatment follow-up are essential to improve outcomes in this vulnerable population.

## Figures and Tables

**Table 1:** Epidemiological, clinical, and laboratory characteristics of the patients with visceral leishmaniasis, according to their immune status.

Characteristics	Total (*n* = 144)	Immunocompetent (*n* = 102)	Immunocompromised (*n* = 42)	*p*-Value
Age (years)	41.8 ± 26.5	35.3 ± 25.8	57.5 ± 21.2	<0.001
Male sex	85 (59%)	66 (65%)	19 (45%)	0.031
Greek nationality	123 (85%)	87 (85%)	36 (86%)	0.95
Residential status				0.32
Urban	56 (39%)	37 (36%)	19 (45%)	
Rural	88 (61%)	65 (64%)	23 (55%)	
Clinical symptoms				
Fever	137 (95%)	97 (95%)	40 (95%)	0.97
Weakness	87 (60%)	57 (56%)	30 (71%)	0.083
Rigors	65 (45%)	43 (42%)	22 (52%)	0.26
Perspiration	48 (33%)	37 (36%)	11 (26%)	0.24
Anorexia	45 (31%)	32 (31%)	13 (31%)	0.96
Loss of weight	57 (40%)	38 (37%)	19 (45%)	0.37
Cough	39 (27%)	27 (26%)	12 (29%)	0.80
Abdominal pain	20 (14%)	14 (14%)	6 (14%)	0.93
Arthralgia	18 (12%)	9 (9%)	9 (21%)	0.038
Diarrhoea	17 (12%)	8 (8%)	9 (21%)	0.022
Vomiting	14 (10%)	7 (7%)	7 (17%)	0.12
Headache	12 (8%)	10 (10%)	2 (5%)	0.51
Rhinitis	9 (6%)	8 (8%)	1 (2%)	0.28
Pharyngalgia	6 (4%)	6 (6%)	0 (0%)	0.18
Expectoration	5 (3%)	5 (5%)	0 (0%)	0.32
Dysuria	5 (3%)	4 (4%)	1 (2%)	>0.99
Low back pain	3 (2%)	3 (3%)	0 (0%)	0.56
Physical findings				
Splenomegaly	126 (88%)	92 (90%)	34 (81%)	0.13
Hepatomegaly	77 (53%)	63 (62%)	14 (33%)	0.002
Lymphadenopathy	37 (26%)	30 (29%)	7 (17%)	0.11
Pallor	36 (25%)	27 (26%)	9 (21%)	0.53
Rash	14 (10%)	10 (10%)	4 (10%)	0.96
Peripheral oedema	11 (8%)	5 (5%)	6 (14%)	0.08
Bleeding	8 (6%)	6 (6%)	2 (5%)	>0.99
Ascites	2 (1%)	2 (2%)	0 (0%)	>0.99
Jaundice	1 (1%)	1 (1%)	0 (0%)	>0.99
Laboratory findings				
Anaemia	122 (85%)	85 (83%)	37 (88%)	0.47
Thrombocytopenia	111 (77%)	79 (77%)	32 (76%)	0.87
Leukopenia	104 (72%)	72 (71%)	32 (76%)	0.50
Pancytopenia	84 (58%)	55 (54%)	29 (69%)	0.094
Elevated inflammatory markers	139 (97%)	98 (96%)	41 (98%)	0.65
Polyclonal hypergammaglobulinemia	101 (70%)	72 (71%)	29 (69%)	0.85
Elevated transaminases	70 (49%)	51 (50%)	19 (45%)	0.60
Antinuclear antibodies	50 (35%)	33 (32%)	17 (40%)	0.061
Unknown	81 (56%)	57 (56%)	24 (57%)	
Rheumatoid factor	28 (19%)	20 (20%)	8 (19%)	0.25
Unknown	80 (56%)	52 (51%)	28 (67%)	
Low complement level	18 (12%)	13 (13%)	5 (12%)	0.82
Unknown	67 (47%)	48 (47%)	19 (45%)	
Hemophagocytosis	7 (5%)	6 (6%)	1 (2%)	0.67
Positive tissue microscopy	68 (47%)	46 (45%)	22 (52%)	0.43
Positive serology	121 (84%)	88 (86%)	33 (79%)	0.25
Positive PCR	71 (49%)	49 (48%)	22 (52%)	0.64
Morbidity indicators				
LOS before diagnosis (days)	4.0 (2.0–7.0)	4.0 (2.0–7.0)	4.0 (2.0–8.0)	0.75
Total LOS (days)	9.0 (7.0–15.0)	9.0 (7.0–13.0)	11.0 (7.0–18.0)	0.16
Symptoms duration (days)	25.0 (13.5–45.0)	22.0 (13.0–44.0)	30.0 (20.0–48.0)	0.087
Fever duration (days)	17.0 (10.0–30.0)	15.0 (10.0–30.0)	27.0 (13.0–32.0)	0.11
Fever level				0.54
≤38 °C	10 (7%)	7 (7%)	3 (7%)	
38–39 °C	45 (31%)	29 (28%)	16 (38%)	
>39 °C	73 (51%)	56 (55%)	17 (40%)	
Unknown	9 (6%)	5 (5%)	4 (10%)	
No fever	7 (5%)	5 (5%)	2 (5%)	
Died within 12 months of VL	7 (5%)	3 (3%)	4 (10%)	0.095

Data are presented as mean ± standard deviation or median (interquartile range) for continuous variables, and frequency count (%) for categorical variables. PCR, Polymerase Chain Reaction; LOS, length of stay; VL, visceral leishmaniasis.

**Table 2:** Factors associated with treatment response in 144 patients with visceral leishmaniasis.

Risk Factor	Cure (*n* = 130)	Failure (*n* = 14)	*p*-Value
Age (years)	40.6 ± 26.2	52.6 ± 28.1	0.11
Male sex	77 (59%)	8 (57%)	>0.99
Immunocompromised	34 (26%)	8 (57%)	0.027
Asplenia	2 (2%)	0 (0%)	>0.99
HIV infection	3 (2%)	2 (14%)	0.074
Solid tumour	3 (2%)	0 (0%)	>0.99
Leukaemia	0 (0%)	3 (21%)	<0.001
Lymphoma	1 (1%)	0 (0%)	>0.99
Chemotherapy	1 (1%)	1 (7%)	0.19
Radiotherapy	1 (1%)	1 (7%)	0.19
Autoimmune disease	25 (19%)	4 (29%)	0.48
Receipt of immunosuppressants	20 (15%)	3 (21%)	0.70
Receipt of corticosteroids	16 (12%)	3 (21%)	0.40
Chronic kidney disease	6 (5%)	0 (0%)	>0.99
Liver cirrhosis	1 (1%)	0 (0%)	>0.99
Diabetes mellitus	17 (13%)	1 (7%)	>0.99
Obesity	12 (10%)	2 (18%)	0.35
Intravenous drug user	1 (1%)	0 (0%)	>0.99
Anaemia	109 (84%)	13 (93%)	0.70
Leukopenia	95 (73%)	9 (64%)	0.53
Thrombocytopenia	98 (75%)	13 (93%)	0.19
Pancytopenia	75 (58%)	9 (64%)	0.78
Polyclonal hypergammaglobulinemia	92 (71%)	9 (64%)	0.74
Hemophagocytosis	6 (5%)	1 (7%)	0.52

Data are presented as mean ± standard deviation for continuous variables, and frequency count (%) for categorical variables. HIV: human immunodeficiency virus.

## Data Availability

Access to de-identified individual participant data that underlie the results reported in this article may be granted upon request to the corresponding author, following a review of the data management and sharing plans and upon signing a data-sharing agreement.

## Abbreviations

The following abbreviations are used in this manuscript:

CL	Cutaneous Leishmaniasis
CMV	Cytomegalovirus
HIV	Human Immunodeficiency Virus
HLH	Hemophagocytic Lymphohistiocytosis
IQR	Interquartile range
L-AMB	Liposomal Amphotericin B
LOS	Length of stay
PCR	Polymerase Chain Reaction
VL	Visceral Leishmaniasis
